# Deep learning based retinal hard exudates quantification of optical coherence tomography

**DOI:** 10.1186/s40942-025-00715-z

**Published:** 2025-10-17

**Authors:** Chang Ki Yoon, Hyung Woo Lee, Hyun Woong Kim, Jung Lim Kim

**Affiliations:** 1 Department of Ophthalmology, College of Medicine, Seoul National University, Seoul, Republic of Korea; 2 Department of Ophthalmology, Konkuk University Medical Center, Konkuk University School of Medicine, Seoul, Republic of Korea; 3 Department of Ophthalmology, Good Gang-An Hospital, Eye center, Busan, Republic of Korea; 4 Department of Ophthalmology, Busan Paik Hospital, Inje University College of Medicine, 75 Bokji-ro, Busanjin-gu, Busan, 47392 Korea

**Keywords:** Retinal hard exudate, Deep learning, Volume, Optical coherence tomography

## Abstract

**Purpose:**

To develop a deep learning (DL) model for segmenting retinal hard exudates (HE) from optical coherence tomography (OCT) scans.

**Methods:**

A modified U-Net architecture was trained on manually segmented OCT B-scans of retinal HE. The training set included 1,811 OCT scans from 15 patients with diabetic retinopathy or branch retinal vein occlusion. The model was evaluated using Dice coefficient and accuracy in idependant test set, and its HE area and volume predictions were compared to manually measured HE areas from a previous clinical study. Additionally, a 2D projected image was generated from the 3D structure of the predicted HE.

**Results:**

The DL model achieved a Dice coefficient of 0.721 and an accuracy of 99.9% on the test set. There was a moderate correlation between model-predicted HE volume and area and manually measured HE area from fundus photographs (*R* = 0.589 and 0.618, respectively; both *P* < 0.001). The projected 2D image generated from the model accurately visualized HE details, demonstrating better structural information compared to fundus photographs.

**Conclusion:**

The proposed DL model enables accurate segmentation of retinal HE, offering volumetric data with both horizontal and vertical structural information, which enhances visualization and quantification compared to traditional 2D imaging.

**Supplementary Information:**

The online version contains supplementary material available at 10.1186/s40942-025-00715-z.

## Introduction

Retinal hard exudates (HE) are pathological yellow-white deposits in the retina, commonly associated with various retinal diseases, including diabetic retinopathy, retinal vein occlusion, macular telangiectasia, and age-related macular degeneration [1]. The primary components of HE are thought to be lipid exudates. The pathogenesis of retinal HE is attributed to the breakdown of the blood-retinal barrier and impaired retinal fluid metabolism [2, 3]. Studies have identified HE as an independent risk factor for visual impairment in diabetic retinopathy [4, 5]. 

HE’s amorphous morphology and scattered pattern across the retina pose challenges for precise marking and measurement, often leading to significant inter-grader variability. In contrast, central macular thickness (CMT) is automatically measured by the embedded software in optical coherence tomography (OCT) devices and is frequently used to guide treatment decisions due to its correlation with visual function. However, given the significant impact of HE on visual prognosis, quantitative HE measurement could enhance treatment planning in retinal diseases.

Various strategies and imaging modalities have been explored for HE quantification, including fundus photography, en face OCT imaging, and polarization-sensitive OCT. While most methods provide two-dimensional projected images, OCT volume scans offer three-dimensional data, enabling volumetric analysis. Despite its advantages, manual HE segmentation from OCT scans is time-consuming and prone to low reliability, highlighting the need for automated measurement techniques. In a previous study, we attempted to develop an HE measurement program using edge detection and threshold-based detection techniques applied to OCT images [6]. However, this approach was limited by false-positive segmentation, particularly for hyperreflective regions such as the retinal nerve fiber layer and blood vessels. With the growing application of deep learning in medical image analysis, we aim to develop a neural network model capable of accurately segmenting HE from OCT images.

## Methods

### [Training dataset

The training dataset included OCT scans from 15 patients diagnosed with diabetic retinopathy or branch retinal vein occlusion, where retinal hard exudates (HE) were present. Images were obtained using three different OCT devices: Cirrus HD-OCT (Carl Zeiss Meditec, Dublin, CA), Spectralis OCT (Heidelberg Engineering, Heidelberg, Germany), and Topcon DRI OCT-1 (Topcon, Tokyo, Japan). A total of 1,811 OCT B-scans were collected (Cirrus: 956 scans from 5 patients; Spectralis: 343 from 5 patients; Topcon: 512 from 5 patients). Imaging protocols were as follows: Spectralis: 30° × 20° or 20° × 15° horizontal scans; Cirrus: macula cube 512 × 128 for 6.0 mm × 6.0 mm; Topcon: 3D Macula 512 × 256 for 7.0 × 7.0 mm. OCT image quality was not restricted if the HE lesion was identifiable. Approximately 10% of the OCT scans did not contain visible HE. The test set was composed of data from patients not included in the training set.

All images were de-identified and independently annotated by two masked graders (CKY and HWL). Annotations were performed using Adobe Photoshop (Adobe Systems Incorporated, San Jose, CA, USA) and a touch screen digitizer. Hyper-reflective lesions in OCT were classified as HE based on corresponding fundus photographs to exclude confounding hyper-reflective entities, such as hemorrhages, cotton wool spots, or retinal vessels. Intraclass correlation coefficient (ICC) for the pixel counts of HE was calculated to observe agreement between the independent observers. To ensure a reliability of graders, 90 randomly selected scans out of 900 scans allocated to CKY were segmented by HWL. The ICC of segmented area between the 2 graders was 0.98 (*P* < 0.001).

### [Model training

The backbone deep learning model employed was DUCK-Net, a modified U-Net architecture [7]. DUCK-Net incorporates a novel convolution block (“DUCK block”) that combines multiple kernel sizes for improved feature extraction. This model was selected due to its state-of-the-art performance in medical image segmentation benchmarks. Image augmentations, including horizontal flipping, affine transformations, and color jittering, were applied using the Albumentations library to enhance generalizability. Color jittering was applied exclusively to images, while other augmentations were applied to both images and labels. All images and labels were resized to 352 × 352 pixels.

The model was trained using a dice loss function with a batch size of 4 for 300 epochs [8]. RMSprop optimization was employed with a learning rate of 0.0001. Model implementation was carried out using the TensorFlow framework, and training was conducted on a desktop equipped with an NVIDIA RTX 4090 GPU (24 GB VRAM). Five-fold cross-validation was used to optimize and evaluate model performance. Each fold included one patient per OCT device, ensuring patient-level independence.

### [Data analysis and statistical methods

The model’s performance was evaluated on the independant test set of 150 OCT B-scans. Segmentation accuracy was assessed using the Dice coefficient, comparing model predictions against ground truth labels. Additionally, HE area in each B-scan, calculated as the sum of predicted HE pixels, was compared between model predictions and ground truth using ICC.

[Comparison with Manual HE Detection in a Prospective Study]

Model-predicted HE quantifications were compared to manual HE segmentations derived from fundus photographs. Data for this comparison were sourced from a subset of a previous prospective clinical trial investigating the efficacy of intravitreal dexamethasone implants for HE in diabetic macular edema [9]. Seven participants from the trial were included in this analysis. The HE area measured via manual segmentation of fundus photographs was compared with the HE area and volume calculated from OCT scans using Pearson correlation analysis.

### [Ethics

This study adhered to the tenets of the Declaration of Helsinki and was approved by the Institutional Review Board (IRB) of Inje University Busan Paik Hospital (approval number: [insert number]). The IRB waived the requirement for written informed consent due to the retrospective nature of the study and the anonymization of patient data.

## Results

The deep learning model achieved a Dice coefficient, precision, recall, and accuracy of 0.7211, 0.6984, 0.7454, and 0.9990, respectively, on the independent test set. The mean (± standard deviation) per-scan metrics were 0.703 ± 0.107 for the Dice coefficient, 0.673 ± 0.147 for precision, 0.771 ± 0.143 for recall, and 0.999 ± 0.001 for accuracy. The intraclass correlation coefficient (ICC) of the HE pixel area between the DL model and human graders was 0.965.

**Fig. 1:**
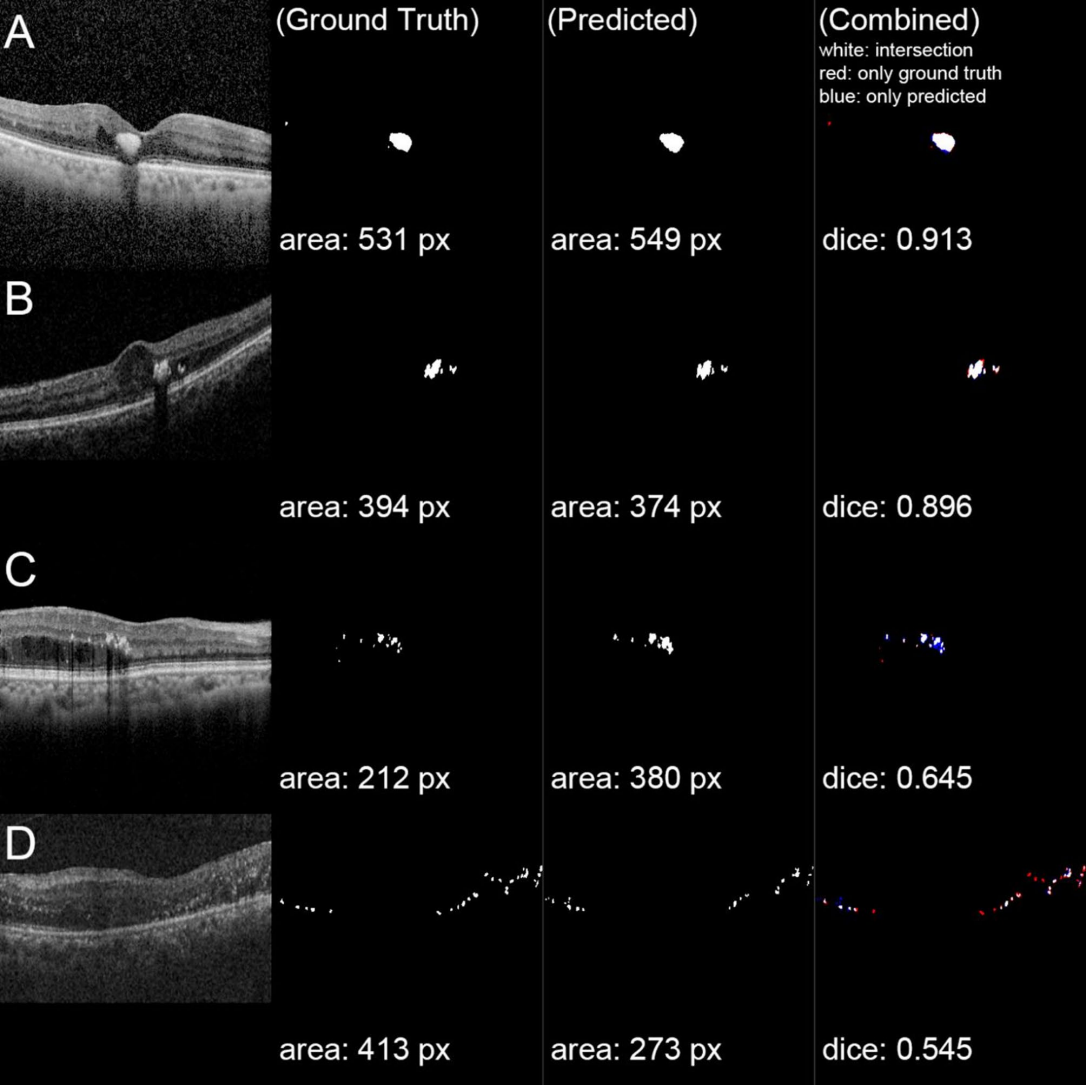
Representative examples of hard exudate (HE) segmentation in the test set of OCT B-scans. The first column shows the OCT B-scan, resized to 352 × 352 pixels; the second column (Ground Truth) displays manually segmented retinal HE; the third column (Predicted) shows HE segmented by the deep learning (DL) model; and the fourth column (Combined) overlays the manually segmented and DL model-predicted HE. In the combined images, white pixels indicate overlap between manual and DL segmentation, red pixels indicate only manually segmented HE, and blue pixels indicate only DL-predicted HE. (**A**) and (**B**) represent cases with high Dice scores, while (**C**) and (**D**) show relatively low Dice scores due to numerous scattered hyperreflective foci

Representative segmentation examples are shown in Fig. 1. Regions segmented by both DL model and graders are shown in white, grader-only regions in red, and model-only regions in blue. While high-contrast HE was consistently identified by both (Fig. 1A and B), the model occasionally missed low-contrast HE and correctly identified ambiguous regions overlooked by graders (Fig. 1C and D).

**Fig. 2:**
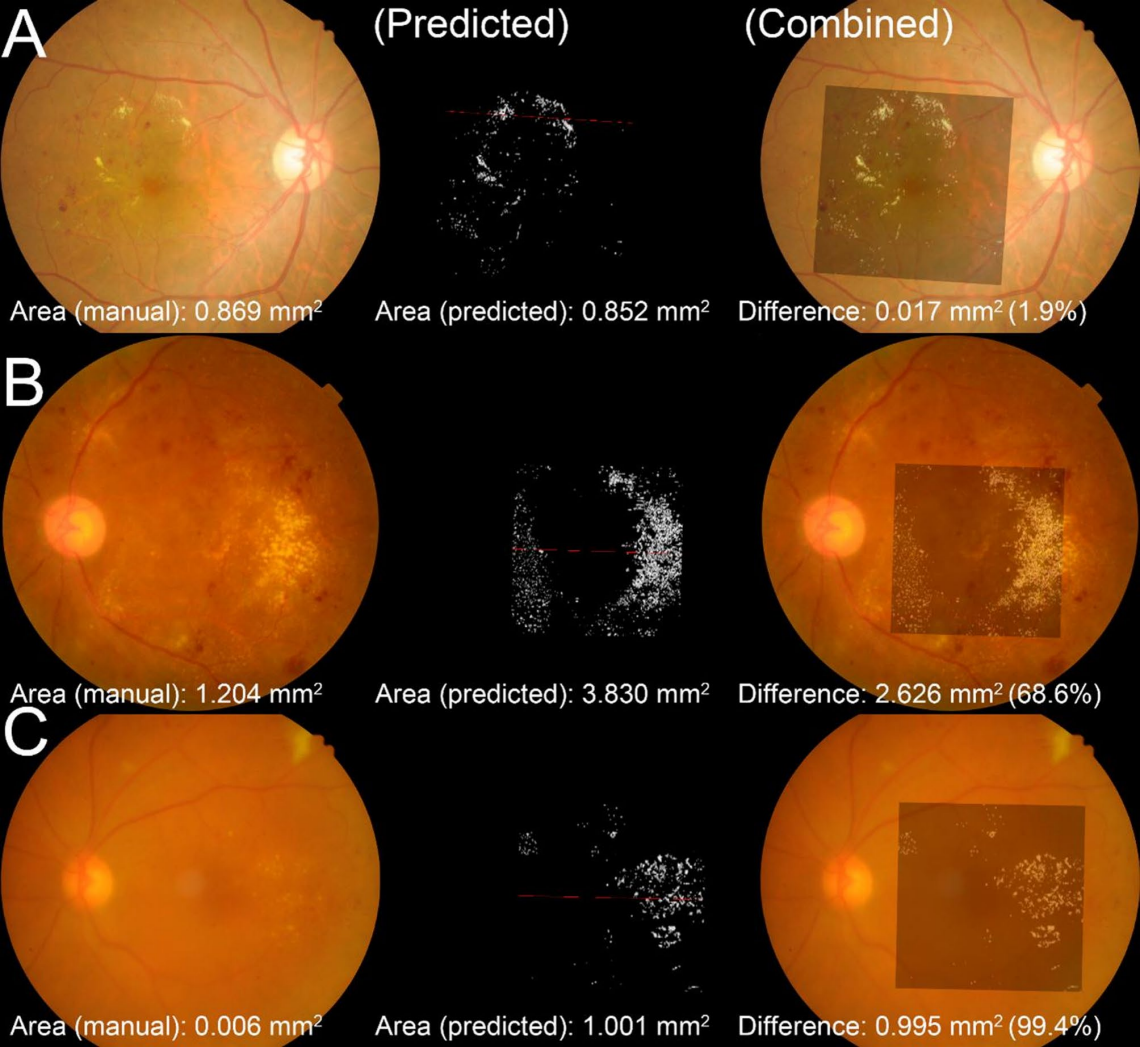
Fundus photo-aligned 2D projection maps of HE derived from volumetric OCT segmentation. The first column shows fundus photographs of diabetic macular edema patients with retinal HE. These images are from a previous clinical trial and are not part of the training or test datasets. The second column (Predicted) illustrates HE segmentation performed on 128 macular volume B-scans, projected onto the fundus photo plane. Pixels with more than three predicted HE voxels along the projection axis are marked in white. The red line indicates the loci of the corresponding OCT B-scans shown in Fig. 3 (**A**, **B**, and **C**, respectively). The third column (Combined) overlays the projected HE image onto the fundus photo with 30% transparency to visualize DL model accuracy. The HE area within a 6-mm-diameter circle is shown at the bottom of each image: (Left) manually segmented HE area, (Middle) DL model-predicted 2D HE area, and (Right) the area difference between the two methods. The area difference is small in case A (1.9%) but significantly larger in cases B and C, suggesting underestimation in manual segmentation. Cases B and C are identified as outliers in Fig. 4, where the solid-line circle corresponds to case B and the dashed-line circle corresponds to case C. The three extreme outliers located between these circles in Fig. 4 are case B data from different visits

**Fig. 3:**
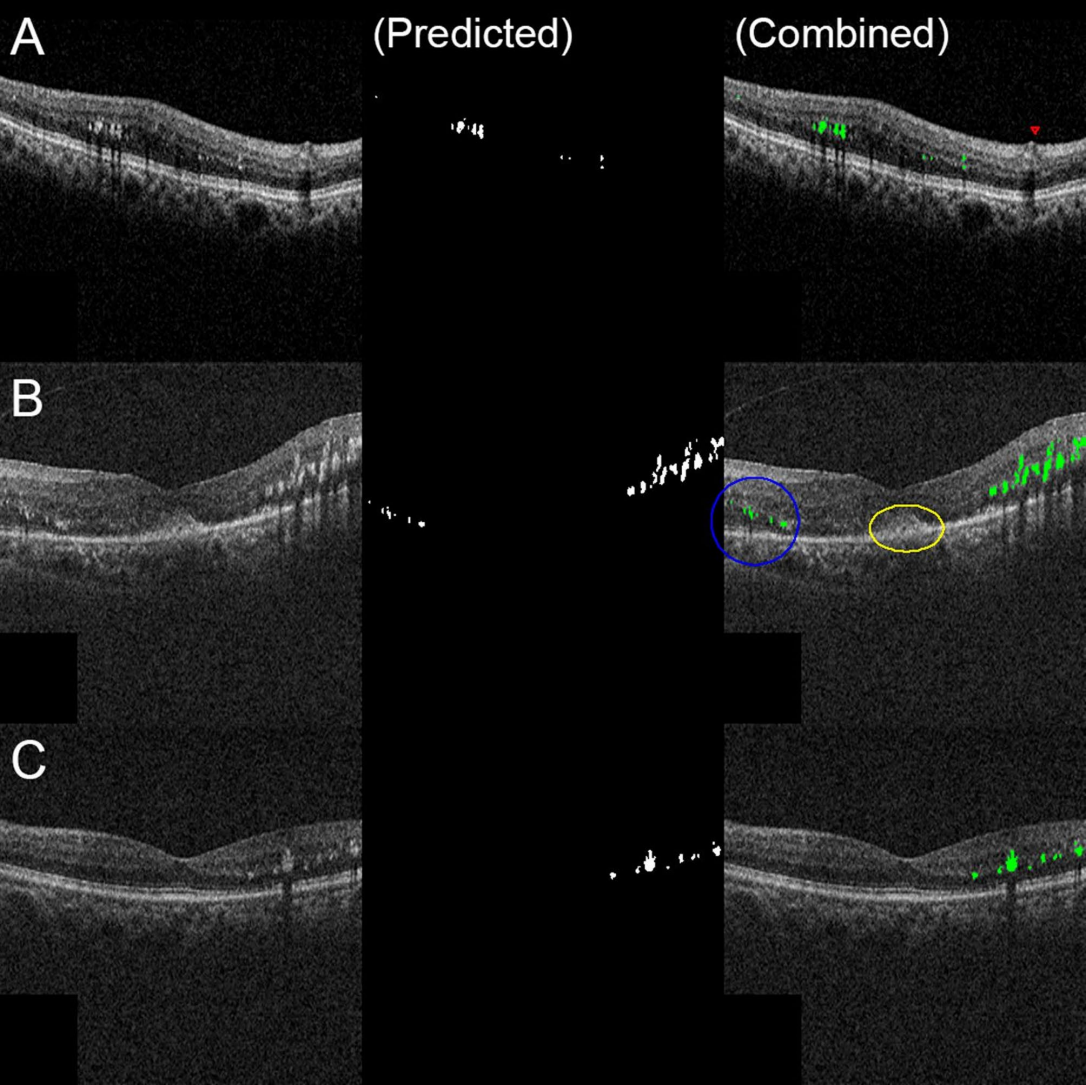
HE segmentation examples from OCT B-scans used in the projected images of Fig. 2. The first column displays the OCT B-scans corresponding to the red lines in the projected 2D images of Fig. 2 (cases A, B, and C, respectively). The second column shows DL model-predicted HE, and the third column presents a combined image of the original OCT B-scan and predicted HE (in green). (**A**) HE is well segmented. The red triangle marks a retinal vessel exhibiting hyperreflective foci with back shadowing, which the DL model correctly excluded from the HE segmentation. (**B**) While HE is not prominent in the nasal macula on the fundus photo, it is detected on the OCT B-scan and correctly segmented by the DL model. Hyperreflective material observed above the retinal pigment epithelium–Bruch’s membrane complex appears yellowish on the fundus photo but is not classified as HE by the DL model due to its unclear characteristics. (**C**) HE is well segmented on the B-scan despite the fundus photo’s haziness

**Fig. 4:**
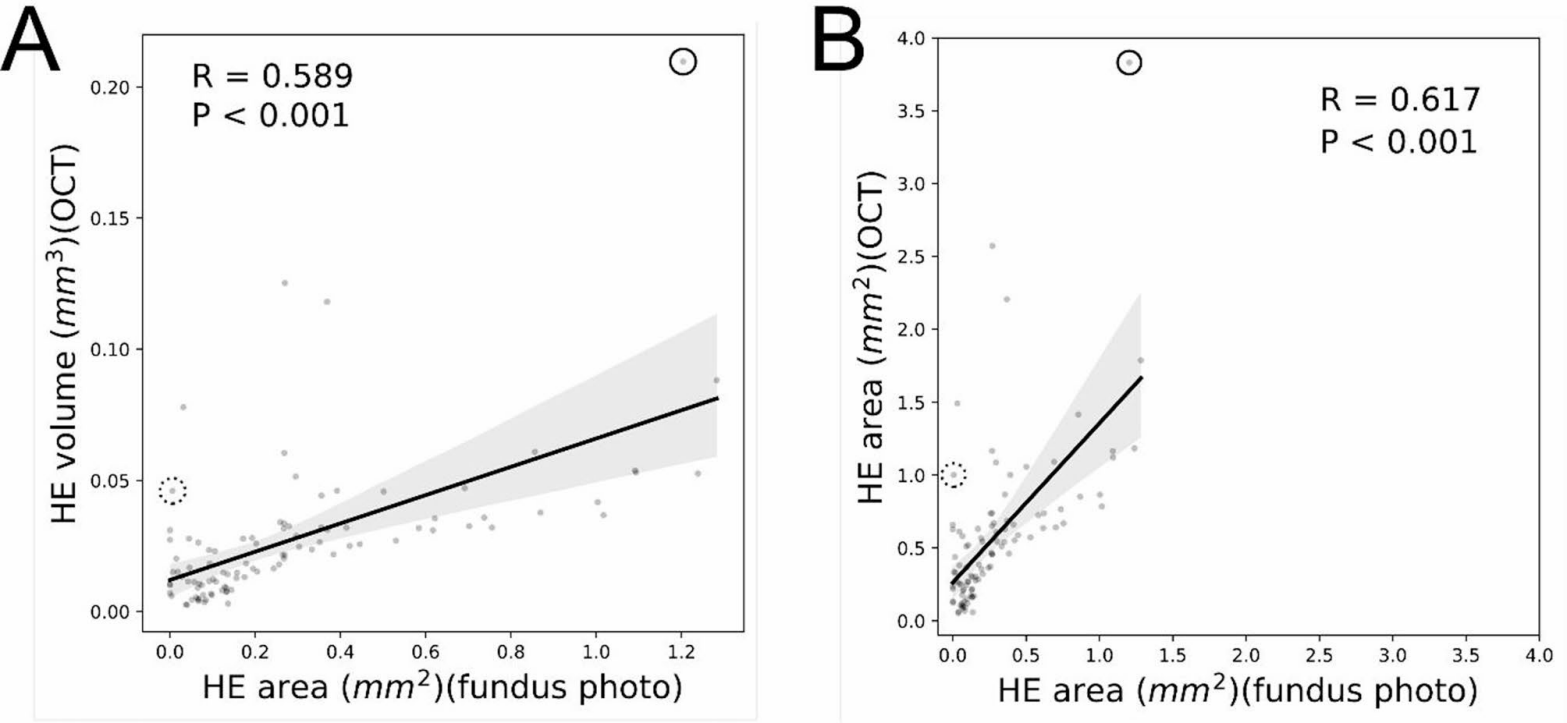
Scatterplot of HE measurements by DL model-based OCT segmentation versus manual segmentation from fundus photos. (**A**) The X-axis represents HE area calculated using manual segmentation of fundus photos, while the Y-axis shows HE volume derived from DL-predicted OCT segmentation. (**B**) The X-axis represents HE area from manual segmentation of fundus photos, and the Y-axis shows the 2D projected HE area from the DL model. Both plots calculate volume and area within a 6-mm-diameter fovea-centered circle. The black trend line and gray confidence intervals indicate the correlation. The correlation coefficient (R) and p-value are noted at the top of the graph. Outliers are circled, with the solid-line circle representing case B and the dashed-line circle representing case C from Figs. 2 and 3

A comparative analysis was conducted between the projected 2D images of HE generated from OCT volume scans and HE areas segmented manually from fundus photographs. For small HE lesions, the DL model demonstrated precise area measurement with minimal discrepancies (Figs. 2A and 3A). Retinal vessels, appearing as hyperreflective dots in OCT scans, were excluded from HE predictions, consistent with manual segmentation (red triangle in Fig. 3A). Notably, HE was visualized in projected OCT images even when obscured in fundus photographs, particularly in the nasal region (Fig. 2B). In this case, manual segmentation underestimated HE, with the DL-predicted HE area being 70% larger than the manual measurement. Additionally, lesions appearing as yellowish regions resembling HE in fundus photographs (Fig. 2B, center) were identified as indistinct hyperreflective material on OCT scans (yellow circle in Fig. 3B). The DL model correctly excluded these lesions as HE. In another instance, media opacity caused by cataracts impeded fundus photo-based HE detection (Fig. 2C). However, the DL model accurately delineated HE due to the sufficient signal-to-noise ratio in OCT scans (Figs. 2C and 3C). In this case, the DL-predicted HE area was 99% larger than the manual measurement. These outlier cases are highlighted in the scatter plots, with solid-line circle corresponding to Figs. 2B and 3B, and dotted-line circle corresponding to Figs. 2C and 3C (Fig. 4).

Correlation analysis showed a moderate correlation between HE volume measurements obtained using the DL model and manual HE area measurements from fundus photographs (*R* = 0.589, *P* < 0.001). Similarly, the HE area from projected OCT images of segmented 3D volumes also exhibited moderate correlation with manual HE areas (*R* = 0.618, *P* < 0.001). Scatter plots illustrating these correlations are presented in Fig. 4.

**Fig. 5:**
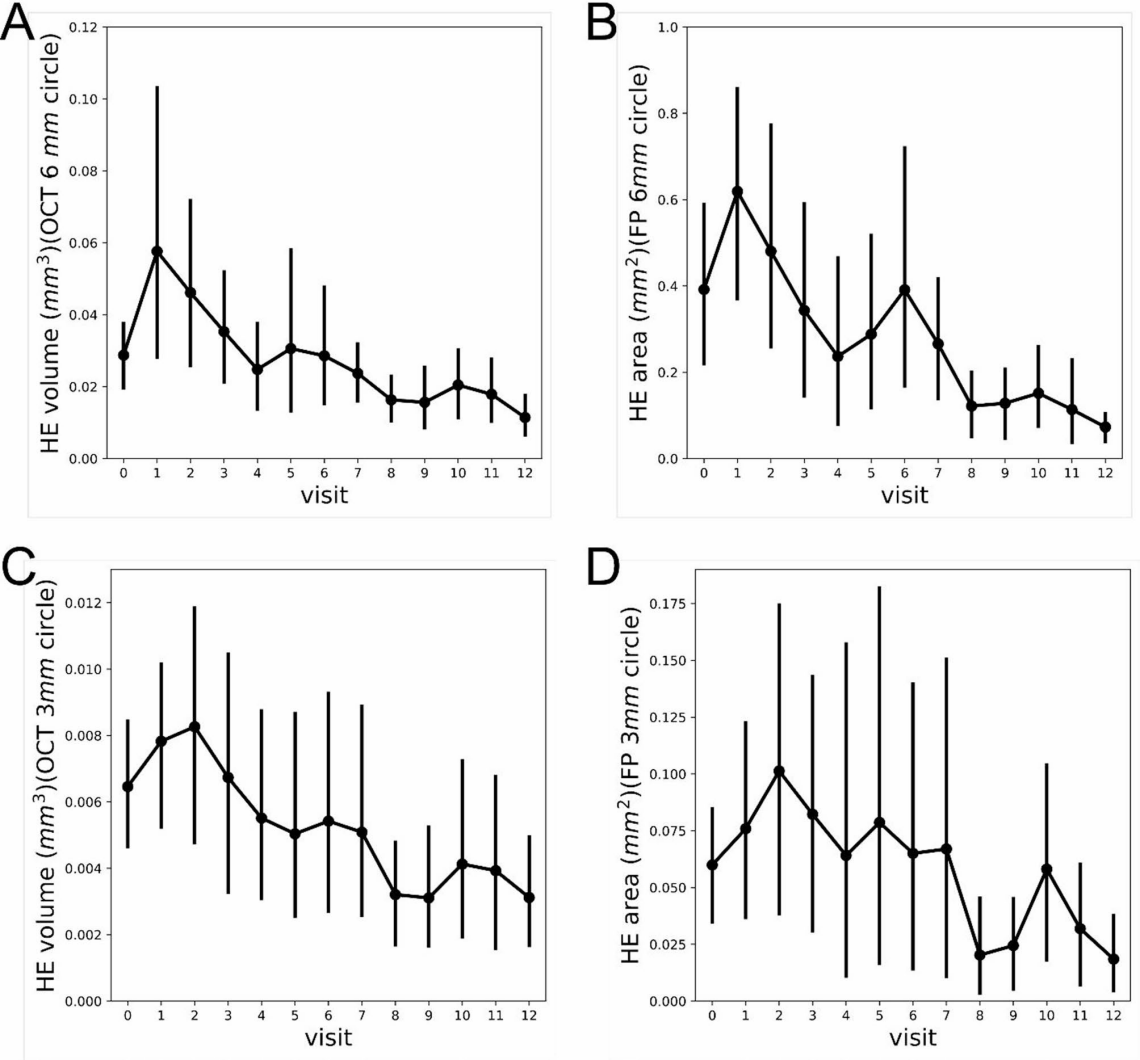
Changes in HE in a subset of clinical trial data: Manual segmentation vs. DL model prediction. (**A**) Average HE volume from DL model-predicted OCT volume scans within a 6-mm-diameter fovea-centered circle. (**B**) Average HE area measured using manual segmentation of fundus photos within the same 6-mm circle. (**C**) Average HE volume predicted by the DL model within a 3-mm-diameter circle. (**D**) Average HE area from DL model-predicted OCT volume scans within a 3-mm circle. Note: This dataset represents a subset of the original data and does not reflect the main study results

Additionally, the average HE volume calculated using the DL model and manually measured HE area from a subset of clinical trial data were plotted over time for each visit. The trends observed in the two methods were similar (Fig. 5A vs. 5B and 5 C vs. 5D).

## Discussion

This study presents a deep learning model for pixel-wise segmentation of retinal hard exudates (HE) in OCT B-scans. This study demonstrates that the DL model predicts HE in OCT with comparable accuracy to retinal specialists, while offering faster and more consistent segmentation. Additionally, the predicted HE volume was validated against human grader-measured HE areas in a clinical trial dataset. The model, trained using data from three different OCT devices, exhibited device-agnostic performance.

To our knowledge, no prior studies have employed the same approach of directly segmenting retinal HE in OCT using a DL model. A closely related study utilized a YOLO-based DL model for detecting HE [10]. However, YOLO generates bounding boxes rather than performing pixel-wise segmentation, making direct comparisons challenging. Nevertheless, the precision and recall of our model surpass the 48.1% and 43.1%, respectively, reported in the YOLO-based study. Rule-based algorithms have also been explored for HE segmentation. For instance, in polarization-sensitive (PS) OCT images of diabetic macular edema (DME) patients, both HE and retinal pigment epithelium (RPE) appeared depolarizing, enabling their differentiation after initial segmentation [11]. This approach showed a correlation coefficient of 0.70 (*P* < 0.001) with manually segmented HE. Another rule-based method, the author participated in developing, segmented hyperreflective lesions by defining regions of interest between the internal limiting membrane (ILM) and RPE based on pixel intensity [6]. This algorithm achieved a moderate correlation coefficient of 0.516 (*P* < 0.001) with manual measurements but often misinterpreted the retinal nerve fiber layer (RNFL), vessels, and RPE as HE (Supplementary Fig. 1). Deep learning-based HE segmentation has also been applied to color fundus photographs, achieving accuracies ranging from 98 to 99.8% [12–15].

Recent applications of deep learning to OCT-based lesion quantification include segmentation of fluid in wet AMD, hyperreflective foci, and geographic atrophy borders. These tools enable rapid, scalable, and objective assessment in both research and clinical settings [16–19]. The integration of deep learning-based imaging biomarkers into clinical workflows enables precise and quantitative assessment of retinal disease burden in real-time during patient consultations. This facilitates personalized treatment decisions at the point of care, a task that would be prohibitively time-consuming and inconsistent if reliant on manual image grading by human experts.

OCT-based segmentation offers distinct advantages over fundus photograph-based measurements. OCT provides volumetric data, enabling accurate quantification of HE. For example, cases 2B and 2 C (Figs. 2 and 3) demonstrate that HE quantities are underestimated in fundus photographs due to their two-dimensional nature. The thicker HE lesions visible in OCT (e.g., temporal HE in Fig. 3B) result in larger volumes than areas derived from fundus photos, a trend also observed in previous studies [11, 20]. OCT excels in detecting small, scattered HE lesions that are often overlooked in fundus photographs, such as those in the nasal and temporal macula (Fig. 2B). OCT also allows for detailed visualization of retinal layers, enabling precise discrimination of HE from other yellow lesions observed in fundus examinations. For instance, a yellow lesion misidentified as HE in fundus photographs was correctly excluded by the DL model based on OCT cross-sections (yellow circle, Fig. 3B). Furthermore, OCT remains effective in detecting HE even under media opacity caused by cataracts or vitreous hemorrhage, conditions commonly associated with diabetic retinopathy [21].

Current DL model is device agnostic on at least three devices. The train dataset is comprised of three different OCT device. In Fig. 1, (A) is examined by DRI OCT-1 (B, C) by Cirrus HD OCT and (D) by Spectralis OCT. In test set, dice coefficient showed no statistical difference among OCT devices.

Despite its strengths, this study has several limitations. External validation was not performed due to the lack of publicly available HE segmentation datasets. However, the model was compared with HE measurements from a separate clinical trial graded by different human graders. Current OCT devices provide macular volume scans ranging from 6 to 10 mm, which is smaller than the field of view in fundus photographs. The development of wide-field OCT can expand the applicability of OCT-based analysis. Additionally, while the volumetric data provided by OCT is advantageous, its clinical correlations with disease severity or outcomes require further investigation.

In this study, we developed a DL model capable of detecting retinal HE in OCT. By leveraging the three-dimensional nature of OCT, this model provides volumetric data that better reflects HE quantity compared to fundus photo-based methods, even under conditions of media opacity. This approach represents a significant advancement in automated HE detection and has potential applications in clinical research and practice.

## Supplementary Information

Below is the link to the electronic supplementary material.

Supplementary Material 1: Supplementary Fig. 1. Comparison of HE segmentation between the current DL model and rule-based algorithms. (A) Fundus photo and 2D projected HE images. From left to right: fundus photo, DL model-predicted HE, and HE segmented by rule-based algorithms. (B-E) OCT B-scans and corresponding segmented HE images. (B) and (C) correspond to the blue line, and (D) and (E) correspond to the red line. The DL model accurately delineated HE contours, whereas the rule-based algorithm failed to detect numerous HE areas. For instance: (B) and (C): The DL model segmented HE clearly, while the algorithm detected only bright pixels. (D) and (E): The DL model detected HE on the left side, while the algorithm misclassified regions of the retinal nerve fiber layer and retinal pigment epithelium as HE.

## Data Availability

No datasets were generated or analysed during the current study.
