# Supplementary figures and images for: Deep learning based retinal hard exudates quantification of optical coherence tomography
Source: Int J Retina Vitreous. 2025 Oct 17;11:114. doi: 10.1186/s40942-025-00715-z (PMC12535103; doi:10.1186/s40942-025-00715-z)

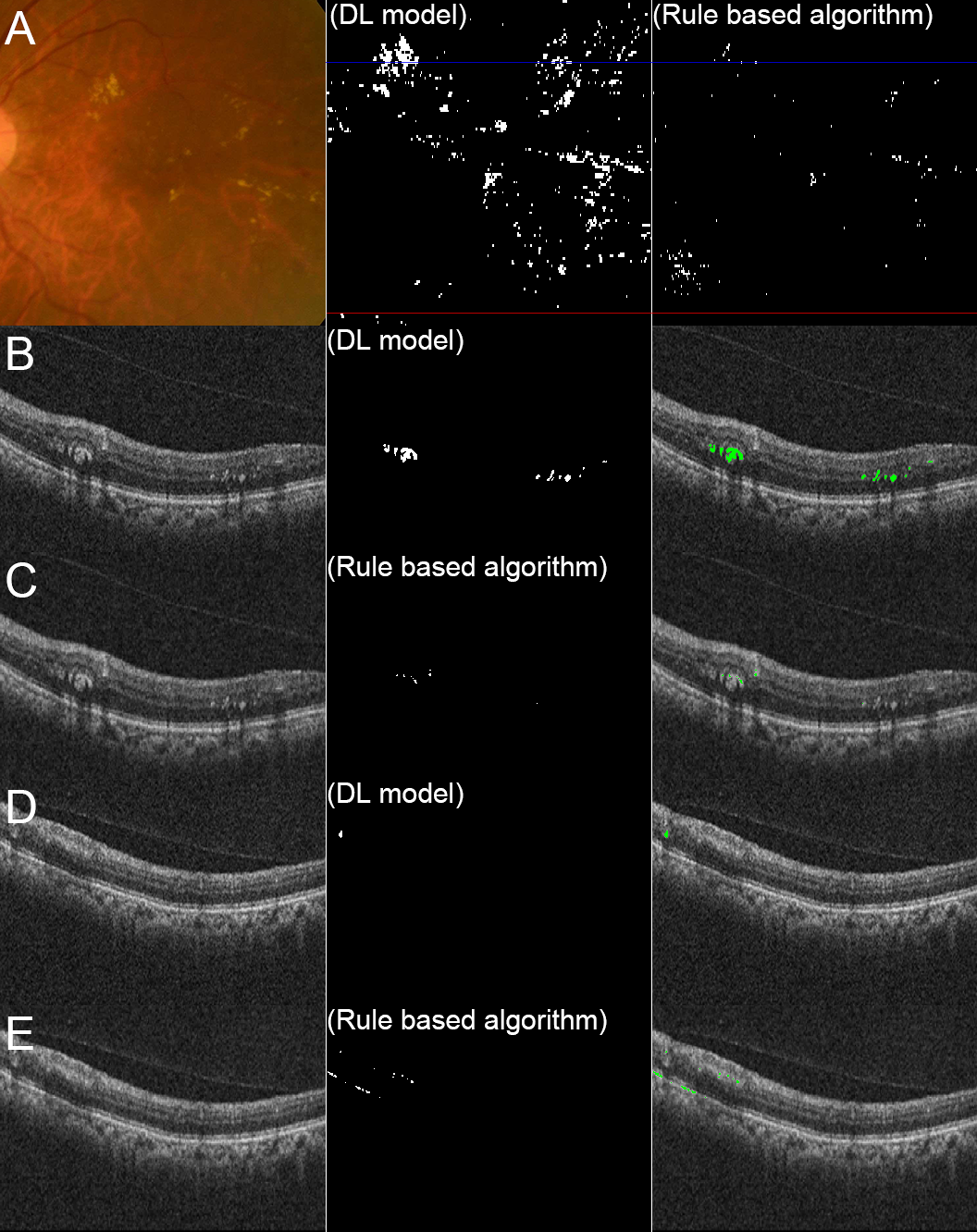

Supplement: Supplementary file 1 — Supplementary Material 1: Supplementary Fig. 1. Comparison of HE segmentation between the current DL model and rule-based algorithms. (A) Fundus photo and 2D projected HE images. From left to right: fundus photo, DL model-predicted HE, and HE segmented by rule-based algorithms. (B-E) OCT B-scans and corresponding segmented HE images. (B) and (C) correspond to the blue line, and (D) and (E) correspond to the red line. The DL model accurately delineated HE contours, whereas the rule-based algorithm failed to detect numerous HE areas. For instance: (B) and (C): The DL model segmented HE clearly, while the algorithm detected only bright pixels. (D) and (E): The DL model detected HE on the left side, while the algorithm misclassified regions of the retinal nerve fiber layer and retinal pigment epithelium as HE. [file 40942_2025_715_MOESM1_ESM.tif]
